# Update on blood‐based biomarkers for chronic liver diseases prognosis: Literature review and institutional experience

**DOI:** 10.1002/jgh3.12667

**Published:** 2021-10-30

**Authors:** Motoh Iwasa, Ryuta Shigefuku, Akiko Eguchi, Yasuyuki Tamai, Yoshiyuki Takei

**Affiliations:** ^1^ Department of Gastroenterology and Hepatology Mie University Graduate School of Medicine Tsu Japan

**Keywords:** biomarker, chronic liver disease, complication, liver cirrhosis, mortality

## Abstract

Liver cirrhosis is the final stage of chronic liver disease (CLD) and is associated with high morbidity and mortality. Various complications such as portal hypertension, ascites retention, hepatic encephalopathy, and hepatorenal syndrome deeply affect patient outcome. The most common tools to predict the outcome of a CLD patient include the following: assessing severity of portal hypertension; scoring systems such as the model of end‐stage liver disease and Child–Pugh score and blood biomarkers related to complications and/or survival rate. In this article, we summarize recent studies of noninvasive markers for predicting impending complications related to CLD and discuss the clinical value of currently available blood biomarkers based on evidence from the literature. In addition, noninvasive blood biomarker assays for different prognostic functions were validated on 113 liver cirrhosis patients at our institution using Kaplan–Meier curve analysis to confirm that these markers can satisfactorily predict CLD‐related patient death.

## Graphical abstract

The possibility of quantifying biomarkers for the prediction of chronic liver diseases with complication. Biomarkers indicate the condition of complication and some biomarkers can be used for the prediction of survival.

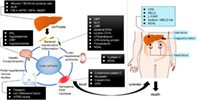

## Introduction

Patients with chronic liver disease (CLD) navigate consecutive liver conditions, including chronic hepatitis, liver cirrhosis (LC), and liver failure; in addition, these patients have an elevated risk of developing hepatocellular carcinoma (HCC).^1^ Various complications such as portal hypertension (PH), ascites retention, hepatic encephalopathy (HE), and hepatorenal syndrome appear in advanced liver disease. Acute‐on‐chronic liver failure (ACLF) is another form of advanced liver disease, which presents acutely with multiple organ failure.^2^ Careful prognostic evaluation and precise management of CLD patients are crucial to reducing the risk of mortality. Although liver biopsy and hepatic venous pressure gradient (HVPG) measurements can provide efficient prognostic evaluation of CLD patients, their application is limited because of the inherent invasiveness of the procedures.^3^ Child–Pugh score (CPS) or model of end‐stage liver disease (MELD) scores generated with multiple factors and variables are used in the assessment and prognosis of cirrhosis and decompensated cirrhosis, respectively.^4^ Recently, the albumin–bilirubin (ALBI) grade was established as an objective hepatic function reserve estimation parameter and is being used instead of CPS.^5^ However, the predicted rate of survival is also greatly influenced by the concomitant stage of HCC or a multitude of other complications that occur in CLD.

In recent years, continuous efforts have been made to investigate the prognostic value of blood biomarkers for CLD patients, including those with HCC, and promising results have been reported.^6^ Here we describe our institution's use of noninvasive blood biomarkers related to different prognostic functions, including the rate of survival and the development of various complications, and present a comprehensive review of the literature.

## Prognostic biomarkers of survival for CLD

Several blood biomarkers have been developed to predict the causal mortality of CLD. CPS and MELD scores are widely used in the prognosis of patients with LC of varying stages.^4^ In addition, serum albumin as a common laboratory parameter is found to be decreased in LC patients and can be utilized for prognostic stratification of LC patients.^7^ Indeed, long‐term human albumin infusion has been found to prolong the overall rate of survival in patients with decompensated cirrhosis.^8^ Albumin synthesis is reflected by the level of branched‐chain amino acids (BCAAs), so patients with a BCAA‐to‐tyrosine ratio <4 have more deleterious events including liver failure and the hastening of death.^9^ Oral BCAA supplementation leads to a reduction in the occurrence of death from any cause in patients with LC^10^, ^11^, ^12^ and improves the oxidized state of serum albumin,^13^ although a recent review showed that BCAA did not have any effect on mortality, quality of life, or nutritional parameters in HE patients.^14^ A low serum albumin and/or BCAA level is considered a physiological hallmark of LC, and the treatment for hypoalbuminemia is now recommended in the guidelines for the treatment of LC in many countries.^15^, ^16^

The ALBI score has recently been reported to objectively estimate liver function status in patients with HCC.^17^ In addition, the ALBI score is used in the prognostic evaluation of various disease states: gastrointestinal bleeding in CLD, ACLF, HCC (treated with sorafenib), HCC (hepatectomy), and transjugular intrahepatic portosystemic shunt creation.^18^, ^19^, ^20^, ^21^, ^22^

Liver‐type fatty acid binding protein (L‐FABP) is an endogenous antioxidant protein that is expressed primarily in the liver but also in the proximal tubular epithelial cells within the kidney. L‐FABP level derived from kidney is elevated in the urine due to renal tubular injury episodes and is therefore used as an established marker of several kidney diseases including acute kidney injury (AKI) and chronic kidney diseases.^23^ In the normal liver environment, L‐FABP is a key regulator of fatty acid metabolism.^24^ Moreover, immunohistochemistry performed on liver sections showed L‐FABP to be expressed in human HCC tissue and associated with vascular endothelial growth factor A expression.^25^ We have reported the prognostic ability of serum L‐FABP level and its correlation with functional measures of the liver in CLD patients, including CH, LC, and HCC.^26^

In general, the level of liver fibrosis predicts liver‐related complications and survival in patients with CLD. Noninvasive tests for liver fibrosis (fibrosis index based on four factors [FIB‐4], aspartate aminotransferase to platelet ratio index [APRI], and Wisteria floribunda agglutinin positive Mac‐2 binding protein [WFA+‐M2BP]) can predict the survival rate of CLD patients.^27^, ^28^, ^29^ These tools may assist physicians in providing a more accurate prognosis at the early stages of the disease.

LC patients often have dilutional hyponatremia due to altered vascular hemodynamics. Systemic arterial vasodilation leads to the release of antidiuretic hormone, which, in turn, induces dilutional hyponatremia. Moreover, profound hyponatremia is frequently associated with severe complications in LC, including ascites, HE, and hepatorenal syndrome.^30^ Several studies have shown that hyponatremia is a strong predictor of early mortality, independent of the MELD score. Owing to an inherent weakness with MELD scores taken in isolation, a combined MELD–Na value has been developed and validated.^31^

## Prognostic biomarkers of LC complication

Several blood biomarkers have been proposed to predict the developmentof LC complications, such as renal insufficiency, PH, HE, bacterial infection, sarcopenia/cachexia, and mortality.We summarize the biomarkers used for assessing each pathophysiology in LC.

### Predicting the renal injury

Many molecules are released via various mechanisms during renal tubular injury in blood and urine. Urinary kidney injury molecule‐1 (KIM‐1) and L‐FABP are released from the proximal tubule, while neutrophil gelatinase‐associated lipocalin (NGAL) is released from the distal tubule.^32^ LC patients with AKI suffer from a mixture of structural (acute tubular necrosis) and functional (pre‐renal azotemia, hepatorenal syndrome) etiologies of renal dysfunction^33^ associated with mortality.^34^

Sharawey *et al*. evaluated the relevance between LC patients with ascites and normal serum creatinine levels and found that serum cystatin C was a predictor of hepatorenal syndrome development.^35^ AKI in patients with ACLF is distinctly relevant to that in patients with CLD. Cystatin C was also a predictor in the development of ACLF.^36^ NGAL, a kidney differentiation inducer and kidney protector, is a binding partner with matrixmetalloproteinase‐9 and has been established as a clinical biomarker for AKI.^37^ We have reported that patients with serum NGAL ≤119 ng/mL had significantly longer rates of survival compared to patients with serum NGAL >119 ng/mL.^38^ Using urine samples, Lei *et al*. reported that urinary KIM‐1 and NGAL could aid in the early diagnosis of AKI secondary to decompensated cirrhosis,^39^ and Verna *et al*. evaluated urinary NGAL for the differential diagnosis of their renal status as well as risk stratification for mortality in LC patients.^40^

### Predicting PH

PH is the main complication of CLD, being the cause of important life‐threatening events including the development of ascites or variceal bleeding. Patients with cirrhosis with a HVPG >10 mmHg have clinically significant PH and are at risk of complications.^3^, ^41^

The von Willebrand factor was reported as a novel noninvasive predictor of PH.^42^, ^43^, ^44^ The von Willebrand antigen‐to‐platelet ratio is a convenient measure to diagnose clinically significant PH independently of CPS.^45^ Copeptin is a stable cleavage product of the arginine vasopressin (AVP) precursor and is equimolarly secreted with AVP. A high serum copeptin concentration predicts survival in hospitalized LC patients and is shown to be associated with short‐term survival in ACLF.^46^, ^47^ Previous studies have shown that high serum copeptin levels associated with hemodynamic changes, such as PH and a HVPG above 12 mmHg^48^ and serum copeptin levels, strongly correlated with variceal bleeding, resulted in the prediction of dangerous PH using copeptin.^49^ We also reported serum copeptin level as a predictor for ascites retention, HE, and portosystemic shunt formation associated with PH.^50^

### Predicting HE

LC patients present with osmoregulation abnormalities in the brain caused by ammonia (NH_3_) hypermetabolism. This NH_3_ hypermetabolism results in astrocyte enlargement and cerebral edema and is thus deeply involved in the pathogenesis of HE.^51^, ^52^, ^53^ Furthermore, hyponatremiais is reported to act as an exacerbating factor for cerebral edema and HE because it reduces osmotic pressure.^54^, ^55^ We have reported that the covert HE group has lower serum sodium, higher serum C‐reactive protein (CRP), and higher NH_3_ levels than the no‐HE group. In addition, covert HE and elevated blood NH_3_ were factors contributing to the development of overt HE.^56^

Zinc plays a key role in the detoxification of NH_3_, a risk factor of HE. Miwa *et al*. reported that zinc deficiency (<60 μg/dL) predicts the development of overt HE and mortality, independently of liver function reserves, using multivariate analyses.^57^

### Predicting the infection

Bacterial translocation (BT) is an important mechanism in the development of infection in LC.^58^ The most widely used BT parameters are CRP, procalcitonin, bacterial DNA, endotoxin or lipopolysaccharide, lipopolysaccharide binding protein (LBP), soluble CD14, calprotectin, bactericidal/permeability increasing protein, and an antibody against to capsular polysaccharide.^58^, ^59^, ^60^, ^61^ Although CRP is a nonspecific biomarker of inflammation, a number of studies have shown that specific CRP values had favorable prognostic indications in patients with LC.^62^ The neutrophil‐to‐lymphocyte and lymphocyte‐to‐monocyte ratios are also used for the prognosis of LC.^63^, ^64^ NGAL expression is induced by a variety of stimulators including bacterial infection. Several clinical studies have shown that NGAL is associated with the survival rate of LC patients when presenting with bacterial infection and that NGAL is a prognostic factor in ACLF patients.^38^, ^65^ LBP, an acute‐phase protein produced by hepatocytes, is found to be a biomarker capable of predicting the development of severe infection.^66^ The serum LBP levels in LC patients with ascites developing severe bacterial infection were significantly higher than in patients with normal LBP levels.^67^ The concentration of LBP is associated inversely with disease severity scores and outcomes in critically ill LC patients with severe sepsis.^68^

### Predicting the sarcopenia or cachexia

The condition of LC patients is often further complicated because of poor nutritional status due to diet‐related states such as protein‐energy malnutrition and sarcopenia, and is associated with the prognosis of LC.^69^ Serum creatinine is frequently used to evaluate renal function in daily clinical practice; however, the level of creatinine can be affected by muscle mass and hepatic creatine production. The age‐ and sex‐corrected estimated glomerular filtration rate (eGFR)creatinine/eGFRcystatin C ratio was found to be an independent predictor of survival in HCC patients based on CLD.^70^ Myostatin is a cytokine capable of strongly suppressing skeletal muscle growth. The serum concentration of myostatin in LC patients was found to be significantly higher than in healthy patients. Nishikawa *et al*. measured serum myostatin levels in LC patients and found that the overall patient survival rates in those with high myostatin were significantly lower than in those with low myostatin.^71^

Zinc‐α‐2‐glycoprotein (ZAG) is a 41‐kDa glycoprotein synthesized by epithelial cells, as well as adipocytes, and plays a role in lipid metabolism, cell cycling, and cancer progression. Cachexia is a known multifactorial syndrome characterized by systemic inflammation, weight loss, and loss of subcutaneous fat area (SFA).^72^ Serum ZAG has been expected as a biomarker for cachexia.^73^ We also confirmed a negative correlation between serum ZAG levels and SFA in LC patients^74^; therefore, ZAG may be a biomarker as cachexia in LC.

## Institutional experience

To validate noninvasive blood biomarker for different prognostic functions using our cohort, we enrolled 113 LC patients (69 males and 44 females) with a mean age of 67.5 ± 10.9 years. We identified 61 patients who presented with HCC in addition to their underlying LC. The study cohort consisted of patients based on a variety of causative agents including 7 with hepatitis B virus, 56 with hepatitis C virus (HCV), 4 with HCV and alcoholism, 13 with nonalcoholic steatohepatitis, 23 with alcoholism, and 10 with other causes. The Child–Pugh grade showed 41 patients in Class A, 54 in Class B, and 18 in Class C. The study protocol has been previously described in detail.^74^ For each continuous variable, the optimal cut‐off value that maximized the sum of sensitivity and specificity was selected using receiver operating characteristic (ROC) analysis for survival. The cumulative survival rates were estimated using the Kaplan–Meier method, and comparison between groups was done by the log‐rank test. The statistical analyses were performed using GraphPad (GraphPad Software, San Diego, IL, USA). Differences were considered to be significant at *P* < 0.05.

**Figure 1:**
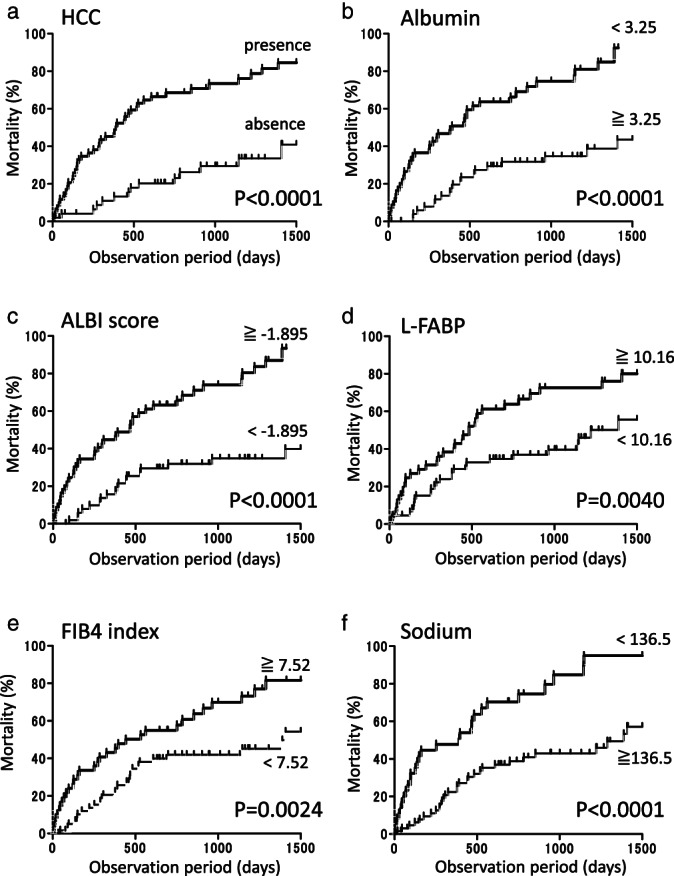
Cumulative mortality rate based on HCC and serum biomarkers: (a) presence or absence of HCC; (b) albumin level; (c) ALBI score; (d) L‐FABP level; (e) FIB‐4 index; (f) sodium level. HCC, hepatocellular carcinoma; ALBI, albumin–bilirubin; L‐FABP, liver‐type fatty acid binding protein; FIB‐4, fibrosis index based on four factors.

Regarding survival, 61 out of 113 LC patients died during the average follow‐up period of 22.0 months (median of 16.0 months). The causes of death were HCC progression in 36 patients, liver failure in 21 patients, and other causes in four patients. There was one patient who received liver transplantation. In our cohort, patient survival was greatly influenced by the presence or absence of HCC (*P* < 0.0001; Fig. 1a), serum albumin levels (*P* < 0.0001; Fig. 1b), ALBI score (*P* < 0.0001; Fig. 1c), serum L‐FABP levels (*P* < 0.0001; Fig. 1d), FIB4 index (*P* = 0.0024; Fig. 1e), and serum sodium levels (*P* < 0.0001; Fig. 1f).

## Conclusions

**Figure 2:**
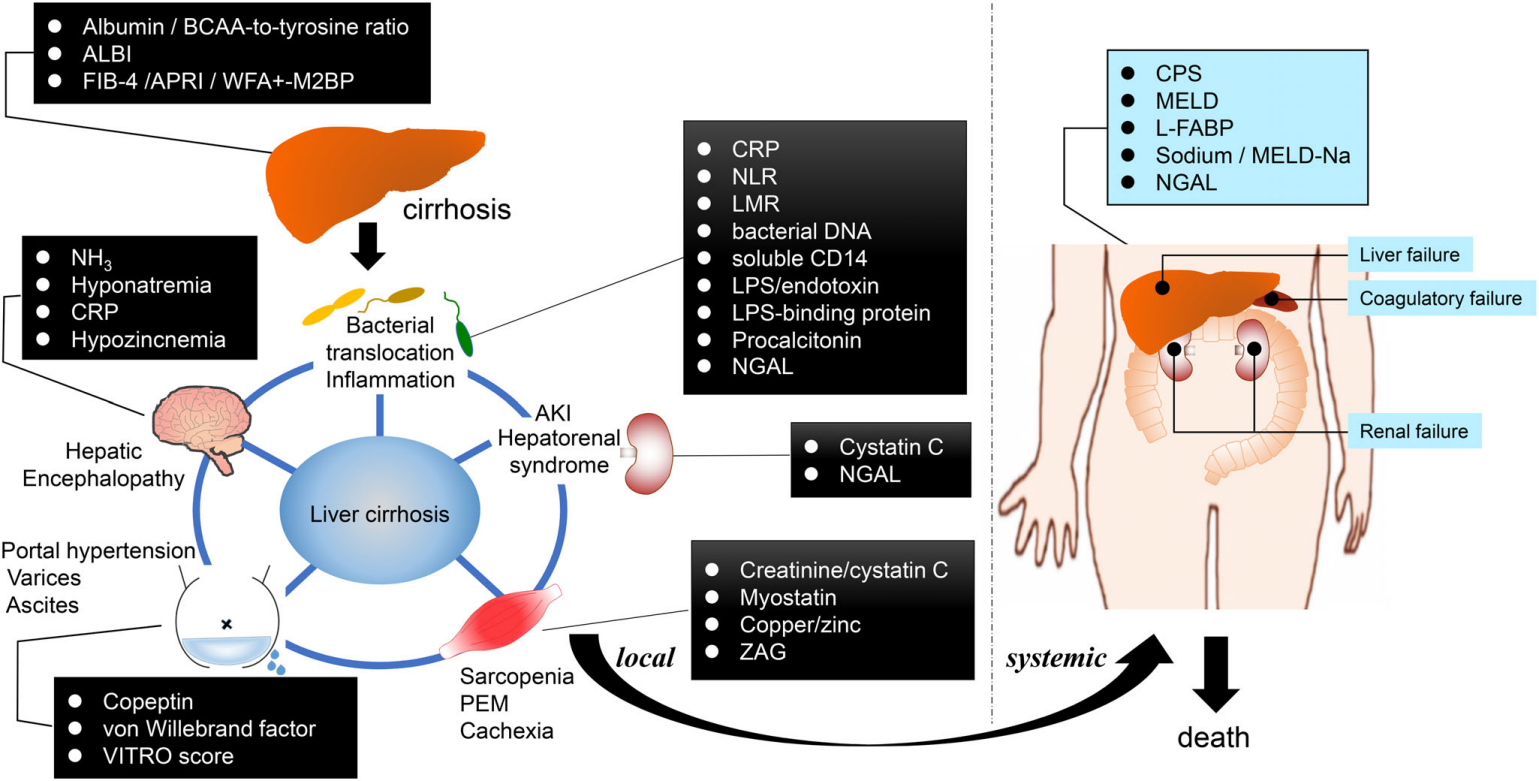
Blood‐based prognostic biomarkers for advanced liver diseases. BCAA, branched‐chain amino acids; ALBI, albumin–bilirubin; FIB‐4, fibrosis index based on four factors; APRI, aspartate aminotransferase to platelet ratio index; WFA+‐M2BP, Wisteria floribunda agglutinin positive Mac‐2 binding protein; CRP, C‐reactive protein; VITRO score, von Willebrand antigen‐to‐platelet ratio score; NLR, neutrophil‐to‐lymphocyte ratio; LMR, lymphocyte‐to‐monocyte ratio; LPS, lipopolysaccharide; AKI, acute kidney injury; NGAL, neutrophil gelatinase‐associated lipocalin; ZAG, zinc‐alpha‐2‐glycoperotein; CPS, Child–Pugh score; MELD, Model for End‐Stage Liver Disease; L‐FABP, liver‐type fatty acid binding protein.

Studies performed for CLD have shown the possibility of quantifying biomarkers for the prediction of CLD (Fig. 2). The utility of biomarkers associated with all‐cause mortality, such as albumin, ALBI score, L‐FABP, FIB‐4, sodium, and NGAL, has been validated in myriad clinical trials. Biomarkers of sarcopenia, inflammation, hemodynamic changes, and AKI are indicators that help characterize the pathophysiology of the disease and its prognosis. Other biomarkers show potential for improving risk stratification. Significant progress in this field will allow new strategies for risk management in patients with CLD to be developed in the near future.
